# Supplementary material for: Characterisation of RSV Fusion Proteins from South African Patients with RSV Disease, 2019 to 2020
Source: Viruses. 2022 Oct 22;14(11):2321. doi: 10.3390/v14112321 (PMC9698603; doi:10.3390/v14112321)
Supplement: Supplementary file 1 [file viruses-14-02321-s001.zip › viruses-1896853-supplementary.pdf]

Supplementary table

Table S1. Summary of F gene sequence data from this study that are available on the GISAID database.

Shortened Sequence name (*Tree)	Full Sequence name (GISAID)	Accession number	Year of detection
RSV25/RSA/2019	hRSV/A/South_Africa/NCV280-25/2019	EPI_ISL_14204587	2019
RSV45/RSA/2019	hRSV/A/South_Africa/NCV496-45/2019	EPI_ISL_14204598	2019
RSV47/RSA/2019	hRSV/A/South_Africa/NCV498-47/2019	EPI_ISL_14204599	2019
RSV48/RSA/2019	hRSV/A/South_Africa/NCV499-48/2019	EPI_ISL_14204600	2019
RSV52/RSA/2019	hRSV/A/South_Africa/NCV500-52/2019	EPI_ISL_14204601	2019
RSV2/RSA/2020	hRSV/A/South_Africa/p5-2/2020	EPI_ISL_14171764	2020
RSV3/RSA/2020	hRSV/A/South_Africa/p6-3/2020	EPI_ISL_14172564	2020
RSV5/RSA/2020	hRSV/A/South_Africa/p7-5/2020	EPI_ISL_14173335	2020
RSV8/RSA/2020	hRSV/A/South_Africa/p8-8/2020	EPI_ISL_14174342	2020
RSV9/RSA/2020	hRSV/A/South_Africa/p9-9/2020	EPI_ISL_14204579	2020
RSV10/RSA/2020	hRSV/A/South_Africa/NCV273-10/2020	EPI_ISL_14204580	2020
RSV11RSA/2020	hRSV/A/South_Africa/NCV274-11/2020	EPI_ISL_14204581	2020
RSV12/RSA/2020	hRSV/A/South_Africa/NCV275-12/2020	EPI_ISL_14204582	2020
RSV13/RSA/2020	hRSV/A/South_Africa/NCV276-13/2020	EPI_ISL_14204583	2020
RSV20/RSA/2020	hRSV/A/South_Africa/NCV277-20/2020	EPI_ISL_14204584	2020
RSV22/RSA/2020	hRSV/A/South_Africa/NCV278-22/2020	EPI_ISL_14204585	2020
RSV24/RSA/2020	hRSV/A/South_Africa/NCV279-24/2020	EPI_ISL_14204586	2020
RSV26/RSA/2020	hRSV/A/South_Africa/NCV317-26/2020	EPI_ISL_14204588	2020
RSV27/RSA/2020	hRSV/A/South_Africa/NCV318-27/2020	EPI_ISL_14204589	2020
RSV31/RSA/2020	hRSV/A/South_Africa/NCV321-31/2020	EPI_ISL_14204590	2020
RSV32/RSA/2020	hRSV/A/South_Africa/NCV322-32/2020	EPI_ISL_14204591	2020
RSV36/RSA/2020	hRSV/A/South_Africa/NCV323-36/2020	EPI_ISL_14204592	2020
RSV37/RSA/2020	hRSV/A/South_Africa/NCV324-37/2020	EPI_ISL_14204593	2020
RSV39/RSA/2020	hRSV/A/South_Africa/NCV325-39/2020	EPI_ISL_14204594	2020
RSV42/RSA/2020	hRSV/A/South_Africa/NCV327-42/2020	EPI_ISL_14204595	2020
RSV43/RSA/2020	hRSV/A/South_Africa/NCV328-43/2021	EPI_ISL_14204596	2020
RSV44/RSA/2020	hRSV/A/South_Africa/NCV495-44/2020	EPI_ISL_14204597	2020
RSV59/RSA/2020	hRSV/A/South_Africa/NCV501-59/2020	EPI_ISL_14204602	2020

*Partial F gene sequences from 2 cases were not uploaded to GISAID
